# Supplementary material for: Homophily in friendship choice during an early life transition: A cohort network dynamic approach
Source: Evol Hum Behav. 2026 Jul;47(4):None. doi: 10.1016/j.evolhumbehav.2026.106888 (PMC13448760; doi:10.1016/j.evolhumbehav.2026.106888)
Supplement: Supplementary file 1 [file mmc3.docx]

# **Homophily in Friendship Choice During an Early Life Transition: A Cohort Networks Dynamic Approach**

**Abstract**

What happens to their friendships when someone moves into a completely new social environment? Moving away from home to attend university is the first major life transition for many, and we studied friendship development in two incoming college cohorts. Using cohort network analysis, we investigated the impact of homophily in a broad range of traits for initial, short-term, and long-term friendship choice. Results revealed that homophily-driven self-segregation occurred within one term for two-thirds of the 16 traits analysed. There was little change once the initial networks had been established, indicating strong selection for homophily at the outset. Since, in a more mobile workforce, major transitions of this kind are now more common, there is a need for further work on these rapid social developments. This may be especially important in contexts where new members of a community (e.g. a business) need to be embedded quickly and where failure to do so can quickly result in loneliness.

**Key Words:** friendship, cohort analysis, network structure, homophily

**Main text:** 7893 words

## 1. Introduction

While mate choice (associated with a romantic partner) and parent-offspring relationships have obvious direct fitness relevance, the fitness benefits of friendships are more indirect. Friendships evolved principally to buffer us against the stresses of living in large social groups, and through that influence our health, longevity and fertility (Dunbar 2025a). Having *any* friends (as opposed to being a social isolate) has a dramatic impact on longevity, the ability to resist and recover from illness and injury, and fertility (see Dunbar 2018, 2025b). This is true for both humans and other social mammals, including primates and equids: social females recover faster from injury, have lower cortisol titres (indicating lower stress levels), live longer, have higher social rank, are more fertile and have higher offspring survival rates (Wittig et al. 2008; Silk et al. 2009, 2010; Cameron et al. 2009; Nuñez et al. 2015), Unlike more conventional adaptive traits such as stature or competitive and problem-solving abilities, however, friendships depend on a reciprocated relationship to provide their benefits. As a result, friendships require considerable time investment, both to establish and to maintain (Dunbar 2018; Hidd et al. 2023). How well these traits gel between individuals (the so-called homophily effect: McPherson et al. 2001; Curry & Dunbar 2011, 2013) influences both the stability and the functional effectiveness of friendships. In this respect, friendships depend on psychological traits that often differ from those that make individuals attractive as mating partners (Dunbar et al. 2024).

This raises questions about the processes that underlie friendship formation and the role these play in the evolutionary process. Conventionally, evolutionary biologists differentiate explanations into four complementary levels – function, mechanism, ontogeny and phylogeny (Tinbergen’s “Four Why’s”: Tinbergen 1963). This is a reminder that it is important to consider mechanisms (the processes that enable fitness functions) when evaluating a trait’s adaptiveness. The mechanisms that produce a function are an important part of the story. Here, we focus on the mechanisms that underpin friendship formation. Of particular importance in this context is the fact that the number of relationships we can maintain at any one time are strictly limited (Dunbar 2020). Hence, when moving into new social environments that offer new friendship opportunities, we are obliged to make decisions about whether to maintain old friendships or abandon them in favour of new ones. In studies of a major life transition (moving away from home to start college), we typically abandon old “home” friends (but not family relationships) in favour of new friendships, while retaining broadly the same number of relationships (Saramäki et al. 2013; Kempnich et al. 2024). How we do this and what traits influence how well we do this then become of central interest.

Baumeister & Leary (1995) proposed that the “need to belong” (the desire to establish and maintain supportive social relationships, akin to Maslow’s [1943] ‘love and belongingness’) is one of our most fundamental evolutionary drives. Being socially well embedded has been shown to have very significant effects on both psychological *and* physical health and wellbeing (Cacioppo et al. 2002; Dunbar, 2025) in ways that will inevitably influence fitness. Feeling socially excluded triggers strong, immediate negative reactions that can have longterm negative effects (Cacioppo & Patrick 2008; Cacioppo et al. 2014; Gere & Macdonald, 2010). For this need to be met, interactions have to be recurrent and generally pleasant. Furthermore, they must occur in a “temporally stable and enduring framework of affective concern for each other’s welfare” (Baumeister & Leary, 1995, p. 497).

The connection between challenging environments and increased efforts to form friendships suggests that strangers entering university together (a phase of uncertainty) are likely to quickly self-organise into a connected community, especially when everyone entering the environment is a stranger (Hechanova-Alampay et al., 2002; Tran, 2016). Such situations involve considerable uncertainty, described by Stillman & Baumeister (2009) as an ‘acute belongingness threat’. In unfamiliar environments, people tend to bond quickly with those who are in close proximity (Berg & Clark, 1986; Hays, 1985; McPherson et al., 2001). The rapid establishment of friendships may be an essential strategy for coping with these kinds of transitions.

Stable friendships are dominated, above all, by homophily (the preference for people who are similar) (Lazarsfeld & Merton, 1954). In their landmark review, McPherson et al. (2001) argued that homophily is one of the most powerful principles governing friendship formation. Similarities can be based on sociodemographic, psychological, or behavioural characteristics, as well as on attitudes and abilities (see also Dunbar 2018). The influence of this broad range of factors has been observed both across all types of relationships (including kin, friends, co-workers, and romantic partners) and throughout the life course. Homophily effects may thus be especially relevant for young adults when navigating the psychologically most turbulent period in life during which they are most actively engaged in the selection and formation of friendships (Kandel et al., 1990). To explore this in more detail, we here focus on the role of homophily in establishing new friendships in a major life transition, that from the home environment to college that typically occurs at age 18 years at the end of high school.

The strongest and most consistent homophily factors identified by McPherson et al. (2001) were sociodemographic: ethnicity, gender, age, religion, and education. Homophily in occupation, social network, behaviours and intrapersonal values were also noted, but appeared difficult to disentangle from the more salient sociodemographic factors. In addition, there is evidence for homophily with regards to personality characteristics (Selfhout et al., 2010) and mental health (e.g., Schaefer et al., 2011). Some similarities can be immediately obvious (e.g., sharing the same age, ethnicity or gender), but many require sustained interaction to identify (e.g., aspects of personality, or political or religious allegiances). Reagans (2011) referred to these as ‘surface’ and ‘deep’ homophily, respectively. He noted that a shared trait’s salience influences its impact in a given situation. Two Germans meeting abroad, for instance, might attribute considerably more significance to their shared nationality than if they met in Germany.

Whether salient or covert, homophily is likely to influence how individuals assemble into groups. Close proximity, or propinquity, may help networks to develop quickly through the provision of opportunities for contact because they allow similar individuals to find each other more quickly, leading to the rapid formation of homogenous subgroups within a larger community (Marsden, 1988; Zipf, 1949). Homophily may also be facilitated by the fact that similar people tend to be drawn to similar places (Verbrugge, 1977) – or, in some cases, are forced into them by pricing and/or housing policies. Many communities thus consist of like-minded individuals, one consequence of which is that neighbourhoods segregate along ethnic lines (Blumenstock et al., 2015; Farley et al., 1994). Many organisations are homogeneous for personality for similar reasons (e.g. King et al., 2017). In other cases, homogeny may be imposed for administrative reasons. Stratification based on age is pervasive in the education system, and higher education specifically, but may also reflect other socio-demographic features (Ekstrom, 1961; Shavit et al., 2007).

While we can legitimately ask what criteria people choose their friends on (friendship choice), we need to recognise that friendships are generally not static arrangements but have temporal durations that vary considerably (Blieszner & Adams, 1992; Roy et al. 2022; Hidd et al. 2023). This instability is exacerbated during major life transitions such as school transitions (Escribano et al. 2023; Hafen et al., 2011) and the post-school transition to college (Roberts & Dunbar 2011; Saramäki et al. 2014; Kempnich et al., 2024). Observing changes in friendship patterns over time recognises this inherent flexibility in human relationships and is especially relevant for studying a group coming together for the first time. Furthermore, when former strangers become friends, we can expect similarity to be continuously reappraised. While ‘surface’ similarity may be the most salient initially, ‘deep’ similarity might only emerge as people get to know one another better over time (Reagans, 2011). Hwang et al. (2012) provide an example of originally influential surface-level homophily fading in favour of deep-level homophily in the context of knowledge transfer between colleagues within an organisation.

In addition to tracing changes in the relative strength of specific homophily effects, longitudinal studies allow us to determine whether homophily plays a more active role at specific times during a community’s development. McPherson et al. (2001) suggested that the influence of homophily generally increases over time. However, Godley (2008) presented evidence that homophily effects can fade over time: in a sample of university students, friendships were initially formed primarily on the basis of similarity in their first year, but in the three years that followed friendships were mostly based on simple propinquity. Similarly, Escribano et al. (2023) reported that friendships in a cohort of secondary school adolescents changed in successive years when friends moved to different classes. Since cross-sectional designs only examine a static snapshot and fail to capture friendship dynamics, studying the effects of homophily on friendship selection longitudinally is thus necessary, as we cannot simply assume that effects observed at one time point are representative of later stages of the affiliation process.

The few studies that have investigated friendships longitudinally have mostly focused on friendships among children (e.g., Graham et al., 1998) or measured changes in friendship intensity, rather than friendship selection (e.g., Hays, 1985). A study by Hafen et al. (2011), for instance, showed that stable friendships among Swedish youth were homophilous for delinquency, intoxication, achievement motivation and self-esteem compared to unstable friendships. There remains, therefore, a gap in the literature when it comes to investigating longitudinal homophily in adult friendships.

Homophily in friendships has been primarily investigated by relying on self-report, which is inevitably prone to biases (Marsden, 1990). In most studies, participants are asked to list one or more meaningful relationships alongside further information (specifying duration, shared characteristics, intensity, etc.), leaving the results vulnerable to both demand characteristics (Orne, 1962) and social desirability response biases (Phillips & Clancy, 1972). Denying socially undesirable characteristics (for example, avoiding ethnically different individuals) or trying to fulfil the researcher’s expectations (e.g., by listing as many friends as a questionnaire allows) can distort a dataset. Furthermore, participants might inadvertently consider their friends to be more similar to themselves than they actually are. Research relying exclusively on self-report might thus yield biased and inaccurate information not only about the participants themselves but also about their nominated friends (Feld & Carter, 2002). In addition, the friendship literature has long remained individual-focused, notwithstanding the fact that friendship dyads are invariably embedded in networks (Felton & Shinn, 1992).

A social network approach addresses all of these sources of bias because it allows us to determine whether alleged relationships are reciprocated (Laursen et al., 2011; Wölfer et al., 2015). Nonetheless, most network studies that investigate homophily in friendships only include few similarity measures in their analyses. Shin & Ryan (2014), for instance, focused solely on 6^th^ graders’ academic performance and examined homophily in academic motivation, engagement, and achievement. Block & Grund (2014) were among the first to address this issue formally, urging other researchers to appreciate the multidimensional nature of networks. Including a “multitude of sociologically relevant dimensions” (Block & Grund, 2014, p. 189) allows different homophily effects to be controlled within one model. This approach can prevent erroneous claims for homophily in ethnicity, for instance, when it might be gender homophily that actually drives friendship formation. To explore which sources of homophily best explain both the initial formation and then maintenance of friendships, it is crucial “to measure homophily simultaneously on multiple characteristics” (McPherson et al., 2001, p. 418).

An important distinction therefore needs to be drawn between the initiation of a friendship and its subsequent maintenance. Most longitudinal studies typically emphasise socialisation effects (becoming more similar to friends over time, especially in behaviour) over selection effects (choosing friends based on existing similarities). This emphasis may be especially important for studies focusing on peer pressure in highly susceptible adolescents, for instance. Most such studies often focus on undesirable socialisation effects in early adolescents, such as alcohol consumption (Huang et al., 2014), smoking (e.g., Mercken et al., 2012), or delinquency (e.g., Weerman, 2011). This narrow focus on the influence of friends on behaviour might, however, not reflect more common issues in adulthood where a more prominent role is often played by the negative consequences of self-segregation based on characteristics over which one has little control (e.g., Phillips, 2006). Hence, there remains a gap in the literature when it comes to investigating longitudinal adult friendship selection processes in a new group.

When investigating how homophily shapes the formation and development of a new community, such as a student cohort, the following three elements are thus central: taking a longitudinal approach, analysing an entire network, and focusing on friendship selection with regards to a broad range of possible sources of homophily. To address these, we studied the development of an emerging network in two successive cohorts of incoming students at a residential college of an elite British university. We recorded these students’ cohort-based friendships several times during their first year, and again towards the end of their time at university, to explore a comprehensive inventory of sixteen possible sources of homophily. While a very wide range of traits can (and do) provide a basis for homophily, to keep the analyses within reasonable bounds we limited our analyses to a set of demographic traits (gender, ethnicity, educational background), personality traits (the Big Five traits of openness, conscientiousness, extraversion, agreeableness and neuroticism), social attitudes (political identity and religious affiliation), mental health indices (depression and anxiety) and network parameters (cohort popularity indexed as the number of friendship nominations received, and cohort activity indexed as the number of nominated friends, as well as the quality and quantity of social relationships before starting at university).

We used SIENA (Simulation Investigation for Empirical Network Analyses: Snijders et al., 2010) software to examine the dynamic interactions between these factors over time. This design enables consideration of a range of homophily factors in one model that incorporated both the network’s structure itself and its longitudinal changes (differentiating between immediate, short-term and longer-term homophily effects). In this way, we can distinguish between what happens in the first phase (when strangers are thrown into an unfamiliar environment and encouraged to bond) versus what happens progressively over time as they get to know each other better and form more stable friendships within a community. It is important to appreciate that a SIENA analysis first identifies the factors that determine network structure at the outset and then identifies what *new* factors, if any, influence subsequent changes in network parameters in addition to the original set of variables.

An incoming cohort of a college provides a particularly suitable setting as the sample size is relatively large (around 120 students) with very clear network boundaries. In addition, colleges provide a unique environment for studying these effects because they are both residences (for all students and some faculty) and the context in which a significant proportion of teaching is conducted. Thus, students sleep, eat and socialise (including playing sports) as well as being taught within a physically and socially bounded community.

Combined with the insights from work on the evolution of student friendship networks (van de Bunt et al., 1999; Zeggelink, 1993), our approach can be formalised in terms of three principal hypotheses: in the socially challenging environment provided by joining a college as a freshman, individuals should quickly (within their first term) form new friendships, with few isolates (H1); preferences for friend formation will be dictated by homophily on one or more demographic, psychological and social traits (H2); and if individuals are able to identify others with whom they share traits relatively quickly, these early friendships should be stable once established, whereas if searching for congenial friends takes many months friendships will continue to change over time (H3). In testing H3, we consider two different time frames: changes across the first year (H3A) and subsequent changes that occur into the second and third years (H3B).

## 2. Methods

### 2.1. Study design and participants

We studied two successive incoming student cohorts (Studies 1 & 2) at one college of an elite British university. Each cohort completed a series of online questionnaires about their friendships at various stages during their time at university, as well as an assessment (T0) of their home friends and family in the September prior to starting university in October. Study 1 comprised three assessments during the students’ first year (at the end of each term in December, March and June: T1, T2 and T3, respectively), with a fourth assessment (T4) towards the end of their’ time in college (2.5 years after the first assessment) designed to examine longer-term changes in cohort network structure. In the light of the stability in both friendships and network structure across the first year at college (see Kempnich et al., 2024) and at the request of the college to reduce participant workload, Study 2 comprised only two assessments in the students’ first year (December and March: T1 and T2), with a third (T4) roughly half-way through participants’ time in college (1.5 years after initial contact).

In Study 1, 90 (46 % women; mean age 18 ± 0.80 SD, range: 18-23) of 121 incoming students (74%) agreed to participate. All contributed to all assessments across the first year of the study (100%) and 80 (89%) contributed to the final assessment (T4). In Study 2, 84 (50.6 % women; mean age 18 ± 0.4 SD, range 17-20) of the 118 incoming students (71%) participated; two students dropped out and one passed away after initial contact, resulting in an overall retention rate 81 participants (96%) across both the year 1 and 2 assessments.

### 2.2. Materials

Both studies used the same online questionnaires (see online *Supplementary Information*). On the first assessment (T0, just before starting at college), participants provided information on their age, gender, ethnicity, nationality, and school background (i.e., whether they had attended a private or a state school). With the exception of age (virtually all participants across both studies were the same age, i.e., 18), and nationality (the overwhelming majority were British: 89% in Study 1 and 88% in Study 2), these characteristics were assessed as possible sources of demographic homophily. In addition, participants were asked to fill out a condensed version of the International Personality Item Pool (mini-IPIP) (Goldberg, 1999). The Mini-IPIP uses only 20 items (four per personality trait: Donnellan et al., 2006) and was chosen for its brevity. It uses a 5-point rating scale ranging from *very inaccurate* (1) to *very accurate* (5) and has been shown to yield reliable results (Baldasaro et al., 2013). The five traits, namely agreeableness (α = 0.78 in Study 1; α = 0.76 in Study 2), conscientiousness (α = 0.57 and α = 0.66, respectively), extraversion (α = 0.83 and α = 0.76, respectively), neuroticism (α = 0.74 and α = 0.72, respectively), and openness (α = 0.73 and α = 0.79, respectively), were assessed as possible sources of homophily for personality. Like the demographic traits, personality traits were assumed to remain fairly stable at this age (McCrae & Costa, 2003) and hence were assessed only once.

Participants were also asked to complete the following two statements to assess their political identity: “I favour political values that are …” and “I am politically …”. Response options were given on a 7-point scale ranging from *strongly socialist* (-3) to *strongly conservative* (3) and *far left of centre* (-3) to *far right of centre* (3), respectively, with both assessed only once. Participants were also asked for their religious affiliation (for details, see *SI*). Since the majority of participants (56% in Study 1, 58% in Study 2) identified as non-religious and most religious participants were Christian (75% and 68%, respectively; see *SI*), participants were grouped as either religious or non-religious in order to test for religion homophily effects. The full set of questions are given in the the online *Supplementary Information.*

Then, on each assessment wave (T1-T4), participants were asked to nominate their fellow cohort friends. On previous studies, we have used a variety of ways of defining a friend, including someone you see with a particular frequency or regularly within a time frame, someone you feel emotionally close to, or a friend defined in a functional way (see Roberts et al. 2014). Our experience has been that these different definitions all elicit the same number of names, and that simply using a generic term such as ‘friend’ (at least in a British context) is generally understood to refer to a specific type of relationship (including a romantic partner).

Participants were also asked to fill out the Patient Health Questionnaire (PHQ-9 made up of nine items: Kroenke & Spitzer, 2002) and the General Anxiety Disorder (GAD-7 made up of seven items: Spitzer et al., 2006). Response options for both were given on a 4-point scale ranging from *not at all* (0) to *nearly every day* (3). These measures were used to assess depression and generalised anxiety disorder, respectively. Depression is the most prevalent mental illness in this age group (Kessler et al., 2003; Kessler & Walters, 1998), especially in a university context (Weitzman, 2004). Generalised anxiety is highly comorbid with depression and affects university students more frequently than other young adults (Eisenberg et al., 2007). These two measures were assessed as possible mental health homophily sources and included in each assessment to acknowledge potential fluctuations over time.

In terms of network measures, *network density* is defined as the proportion of all possible ties in a network that actually occur while *centralization* score is best interpreted as a measure of an individual’s importance as a hub node within a network. There is evidence for homophily with regards to popularity in preadolescent (Logis et al., 2013) and adolescent (Dijkstra et al., 2013) friendship networks. We indexed this is two ways. *Cohort popularity* can be estimated as the network parameter ‘indegree’ (i.e., the number of friendship nominations received from fellow participants). Similarly, *cohort activity* can be determined via network parameter ‘outdegree’ (i.e., the number of friendship nominations by the participant made within the cohort). While these two network parameters are exclusively derived from the friendship patterns within the cohort, it is important to incorporate participants’ relationships outside of this boundary (Wölfer et al., 2015). Therefore, participants’ baseline ego-network (BEN) quality and quantity at T0 before starting university were explored as possible sources of network homophily. During initial contact with participants (T0, in September prior to starting at university in October), they had been asked to list all family members and friends with whom they had meaningful relationships (i.e., their ego-network) and provided emotional closeness ratings (*How emotionally close do you feel to X*, rated on a 1 [neutral] to 10 [very] analogue scale) for each friend (for further details, see Kempnich et al., 2024). The average number of friends prior to starting college (*BEN quantity*) and their mean emotional closeness rating to these (*BEN quality*) were computed to serve as potential network homophily factors outwith the cohort.

In Study 1, participants were asked to list everyone with whom they felt they had some kind of personal relationship (friend, romantic partner, acquaintance, someone with whom they interacted on a *regular* basis) within the college cohort of freshman students (consulting phone, address book and social media contacts as necessary). We did not define these terms, but left individuals to apply commonsense definitions, on the grounds that any variance in definition between individuals is more likely to introduce error variance and hence act conservatively by reducing the significance of any statistical effects. At each following assessment, participants were shown their initial list and asked to add or remove names. In Study 2, an alternative nomination procedure was used to control for recall bias. Instead, at each assessment wave, participants were shown a list of all students in the college in their year (including those who did not themselves participate in the study) and were asked simply to indicate those whom they considered a friend (defined as above). At subsequent assessments, participants were not shown their past choices and were thus not reminded whom they had previously listed.

The amended nomination procedure of Study 2 was chosen for three reasons. First, Study 1’s method had allowed for errors, as participants could list friends that were outside of the cohort boundary or that were difficult to accurately identify as cohort members (due to misspellings, the use of nicknames, or inconsistencies across assessments). Second, there is some evidence that a recognition technique yields more accurate results in larger networks (Neyer, 1997). Third, the stochastic actor-oriented models used to analyse network dynamics require a certain amount of change in the friendship nominations between assessments in order to accurately estimate parameters and hence fit a model (Veenstra et al., 2013). Showing participants their initial friendship lists at subsequent assessments in Study 1 could have produced networks that were too static to generate a useable model.

The data are available in online *SI* files *Study1_Datafile* and *Study2_Datafile.*

### 2.3. Analyses

For the purposes of analysis, all the non-categorical variables were converted into binary scores using the median value for a given trait, separately for each study. The median criterion is thus study cohort-specific, and might vary between the two cohorts. This is a requirement for SIENA modelling (see *SI*).

We first examined the initial cohort networks of both studies cross-sectionally at T1 to evaluate the likelihood of homophily effects. Using one-sample t-tests, we compared the overall proportion of same-trait friends on each of the 16 homophily dimensions with the proportion that reflects nominations based purely on chance for a median-partitioned score (i.e. *p*=0.50). To compare the relative influence of individual sources on friendship within a socioeconomically privileged subset of society (Scull & Cuthill, 2010), it is necessary to differentiate between baseline homogeneity (based on the composition of the group under study) and actual homophily (preference for bonding with similar others) (McPherson et al., 2001). As a second step, we therefore determined which subgroups within each category displayed homophily by comparing the proportion of same-characteristics friends with the proportion of that characteristic within the cohort as a whole (e.g. 0.80 for *white* students and 0.20 for *non-white* students).

The longitudinal development of friendship patterns was analysed using Simulation Investigation for Empirical Network Analyses (SIENA: Snijders et al., 2010) implemented in R. This software package estimates dynamic actor-driven models for the evolution of networks. It can account for and examine the naturally occurring interdependence between the overall network structure and the characteristics of the individuals making up the network (Steglich et al., 2006). SIENA thus allows assessment of which individual traits (demographics, personality, etc.) influence *changes* in relationship formation over time. Since any selection effects can be interpreted as logistic regression coefficients, we can determine log-odds ratios in order to test specific hypotheses. Thus, an odds ratio of 1.7 with regards to *depression* would, for instance, indicate that participants were *e^1.7^* = 5.5 times more likely to make friends with cohort members in the same half of the depression scale. Conventional *p*-values can then be determined for the odds ratios.

An additional advantage of this approach is the possibility of simultaneously including a large number of homophily factors without affecting a model’s power and fit (Ripley et al., 2019). These control for one another, and hence increase the confidence in any specific homophily effects. Each modelled homophily effect is additionally controlled for by two related effects. The *ego* effect accounts for cohort members of a given characteristic who are particularly active in the network (e.g., female members making lots of friends, but not necessarily discriminating whom they befriend in terms of gender). In contrast, the *alter* effect accounts for cohort members of a given characteristic who are popular (e.g., female members being befriended by many cohort members, irrespective of the latter’s gender). These default controls are reported for completeness’ sake, but are not interpreted as they are unrelated to the main research question of this paper.

SIENA models also include structural controls relating to common relationship processes that are unrelated to the traits under consideration (Ripley et al., 2019). These account for the tendency for network members to form relationships with those who are positioned near to them within the overall network structure (denoted by a negative *outdegree* effect, signalling that friendships are not formed independently of social distance), with those who are already considered a friend (*reciprocity*), and with those who are friends-of-friends (*transitive triplets* and *transitive ties*). Incorporating these controls thus also allows us to differentiate homophily effects from these kinds of underlying relationship dynamics. The SIENA model thus includes a comprehensive range of homophily factors: the two default controls per factor, and the four structural controls listed above.

To differentiate short-term from longer-term effects, we ran each simulation twice for each study. The first pair of resulting models, which we refer to as short-term models, focused on the participants’ first year at university (i.e., T1-T3 in Study 1 spanning six months, and T1-T2 in Study 2 spanning three months). The second pair of models, referred to as the longterm models, additionally included the final assessment (i.e., T4). These models tested for changes in the sixteen different homophily effects. Note that significant effects already identified at T1 do not reappear in the T2/T3 or T4 models.

An overall maximum convergence ratio below 0.25 and all individual convergence t-ratios below 0.1 indicate a well-estimated SIENA model (Ripley et al., 2019). The overall convergence ratios for the short-term models were 0.12 and 0.14 for Study 1 and 2, respectively; the equivalent convergence ratios for the longterm models were 0.17 and 0.13, respectively. Furthermore, all individual convergence t-ratios were below 0.1, so that all requirements were met in all models. The rate parameters of all four models indicated that significant network changes occurred between assessment waves (all *p* < 0.001), a precondition for any following analyses. In addition, we used the Jaccard Index calculated between successive assessment waves as an index of the fraction of stable friendship nominations (Veenstra et al., 2013). These indices should be above 0.30 (denoting at least 30% network stability) between each of the successive networks (Ripley et al., 2019). This condition was met with Jaccard indices of 0.87, 0.89, and 0.37 for Study 1, and indices of 0.47 and 0.54 for Study 2. Lastly, all four structural controls in all four models significantly contributed to the model in the anticipated directions (*p* < 0.001).

2.4. *Ethics*

Formal ethics approval was granted by the University of Oxford Combined University Research Ethics Committee.

## 3. Results

### 3.1. Finding friends within the cohort (Hypothesis H1)

Having spent just a term (i.e., eight weeks) at university, participants in Study 1 nominated an average of 14.31±10.03SD other cohort members as friends on assessment T1, supporting H1 that incoming students quickly form new friendships. Overall, 1288 nominations were made, forming the cohort network depicted in Figure 1 (T1). The ratio of realised to total ties gives a network *density* of 0.16 (i.e. 16% of all possible ties were realised). There were no isolates, as each participant listed, or was listed, as a friend by at least one other cohort member. Overall, 54% of friendship nominations were reciprocated. The possibility of some students being listed as friends more often than others was reflected in the *indegree range*, spanning from 0 to 31. This popularity index refers to the number of received nominations and suggests some variation in relative popularity in the cohort. The *centralization* score was 0.19: since this can range from 0 to 1, this value indicates a relatively flat hierarchical structure in, as might perhaps be expected within a university student cohort.

| **T1** |  | **Total # of ties:**  1288  **Outdegree (*SD):***  14.31 (10.03)  **Reciprocity:**  0.54  **Density:**  0.16  **Centralization:**  0.19  **Indegree Range:**  0-31  **Isolates**:  0 |
| --- | --- | --- |
| **T2** |  | **Total # of ties:**  1398  **Outdegree (*SD):***  15.53 (10.33)  **Reciprocity:**  0.53  **Density:** 0.18  **Centralization:**  0.22  **Indegree Range:**  0-35  **Isolates:**  0 |
| **T3** |  | **Total # of ties:**  1215  **Outdegree (*SD):***  16.42 (9.83)  **Reciprocity:** 0.59  **Density:** 0.18  **Centralization:**  0.25  **Indegree Range:**  0-30  **Isolates:** 0 |

Figure 1. Study 1 cohort networks and statistics after one (T1), two (T2) and three (T3) terms at university respectively. Each node (square) represents a student, and each tie (link between nodes) represents a friendship.

The cohort network for Study 2 (Figure 2) is similar to that for Study 1. At T1, participants nominated an average of 16.61±8.23SD cohort members as friends. Overall, 1304 nominations were made, resulting in a network *density* of 0.20 (i.e. 20% of all possible ties realised). There were just two isolates at T2; 61% of nominated friendships were reciprocated. *Indegree range* spanned 0-40. The *centralization* score was 0.30, indicating a slightly more hierarchical structure than in Study 1.

| **T1** |  | **Total # of ties:**  1304  **Outdegree (*SD):***  16.10 (8.23)  **Reciprocity:**  0.61  **Density:**  0.20  **Centralization:**  0.30  **Indegree Range:**  0-40  **Isolates**:  0 |
| --- | --- | --- |
| **T2** |  | **Total # of ties:** 1256  **Outdegree (*SD):***  15.51 (8.52)  **Reciprocity:**  0.66  **Density:**  0.19  **Centralization:**  0.27  **Indegree Range:**  0-37  **Isolates:**  2 |

Figure 2. Study 2 cohort networks and statistics after one (T1) and two (T2) terms at university respectively. Each node (square) represents a student, and each tie (link between nodes) represents a friendship. At T2, there were two isolates (indicated top left of graph) who neither nominated nor were nominated as a friend by another person.

Taken together, these results show that friendship formation is rapid (in under two months), with very few (<1%) isolates, confirming H1. Friendships are moderately strongly reciprocated, and there is limited evidence for hierarchical structuring, suggesting that dyadic friendships are relatively independent of each other. The differences in number of friends listed in the two studies was minimal despite the slight difference in the definitions used and the different ways of eliciting names.

### 3.2. Self-assortment in friendships (Hypothesis H2)

We first determine whether, by the end of the first term (wave T1), participants nominated a higher proportion of friends with whom they shared a trait than would be expected by chance (defined as p=0.50), and then test whether they chose friends with a particular trait in proportion to the availability of that trait in the cohort. The results are given in Tables 1 and 2.

There was a significant homophily effect on friendship choice in ten of the sixteen traits in Study 1 (Table 1) and nine of the sixteen traits in Study 2 (Table 2), both with respect to alternate preference (expected likelihood p=0.50) and with respect to trait availability (right hand column of tables). In at least half these cases, the statistical significance is very high (p<0.001). In Study 1, participants nominated a higher proportion of same-trait friends than would be expected by chance (i.e., *p*=0.50) on four traits: *ethnicity*, *openness, extraversion* and *depression* (all coded dichotomously). Study 2 participants exhibited significant preferences on six traits: *gender* (male or female), *ethnicity*, *openness, extraversion*, *agreeableness* and *political identity*.

This method of computing ‘shared-characteristic’ proportions has the advantage of allowing direct comparisons between the different characteristics. We therefore conducted a one-way ANOVA, comparing the relative importance of the sixteen characteristics in influencing initial friendship choice. Results confirmed that the relative importance of the considered characteristics shaping the cohort network varied significantly in each study (Study 1: *F*_(8.27, 595.29_*_)_* = 2.74, *p* < 0.001, *η^2^* = 0.037; Study 2: *F*_(9.72, 767.70)_ = 2.62, *p* < 0.001, *η^2^* = 0.032; for unequal variances in both cases). In both studies, Bonferroni-corrected pairwise-comparisons indicate that the tendency to befriend similar cohort members was strongest for *ethnicity*. Participants shared a *white/non-white ethnicity* with their cohort friends (Study 1: 66%±27*SD*), and in addition a shared-trait preference on four other traits (*religion*, *p* = 0.003; *openness*, *p* = 0.009; *anxiet*y, *p* = 0.013; *BEN quality*, *p* = 0.002), with all other differences non-significant. In Study 2, the tendency to befriend similar cohort members was again strongest for *ethnicity* (61%±22), with a shared-trait preference higher on five other traits (*religion*, *p* = 0.040; *educational background*, *p* = 0.009; *neuroticism*, *p* < 0.001; *BEN quantity*, *p* = 0.021; *BEN quality*, *p* = 0.016). Participants further reported a higher proportion of same-trait friendships with cohort members who scored similarly to themselves on the personality trait *openness* in comparison to *neuroticism* (*p* = 0.047).

Splitting each trait by binary subgroups for Study 1 produced evidence for homophily with regards to ten characteristics (see Table 1): *female* participants nominated significantly more friends of the same gender than expected, given the proportion of female students in the cohort. Additionally, participants scoring high on personality traits *extraversion* and *agreeableness* nominated more same-trait friends than cohort proportions would have suggested. Participants identifying as politically ‘left’ also nominated more same-trait friends than cohort proportions would predict. With regards to mental health, participants who were *not* diagnosed with *depression* nominated more same-trait friends than expected. With regards to network parameters, highly *popular* participants befriended those who were similarly more popular than cohort proportions would predict. Note, however, that less popular participants avoided befriending other less popular cohort members. Similarly, highly *active* participants (who had made many friends) befriended more similarly active friends than cohort proportions would predict, while less active participants (with fewer formed friendships) avoided befriending less active cohort members. Lastly, those with large baseline (pre-college) ego-networks (*BEN quantity*) befriended other individuals with large numbers of friends more often than expected given cohort proportions.

Broadly similar results were found in Study 2 (Table 2). *Female* participants nominated significantly more friends of the same *gender* than expected given the proportion of female students in the cohort. Participants scoring *high* on personality traits *extraversion* and *conscientiousness*, as well as participants identifying as politically *left*, nominated more same-trait friends than cohort proportions suggested. With regards to network parameters, highly *popular* participants befriended individuals who were more popular, whereas less popular participants avoided befriending alters similar to them. Similarly, highly *active* participants befriended more similarly active friends, while less active participants befriended fewer same-characteristic friends, than cohort proportions would predict.

### 3.3. Short term stability in friendship dynamics (Hypothesis H3A)

Table 3 summarises the results of analyses of short term changes in network structure generated by SIENA for both studies. In Study 1, the successive waves cover a nine-month time span with three assessments (after one, two, and three terms, hence throughout their entire first year at the university). Study 2 covered a six-month period with two assessments (i.e., one after the first and second terms of the freshman year). Estimates represent log-odds ratios specifying the probabilities of network change. The *t*-value for each effect can be determined by dividing the estimate by its standard error.

Significant positive homophily effects imply subsequent *changes* in friendship which meaningfully contribute to the network-autocorrelation of this variable by affecting the network structure (while the variable itself remained unchanged). There was evidence for homophily shaping the evolution of the network in the short run in one instance in each study. In Study 1, the log-ratio of this effect implies that cohort members were *e*^0.30^ = 1.3 times more likely to befriend those of the same gender, while in Study 2 cohort members were *e*^0.47^ = 1.6 times more likely to befriend those who scored similar to themselves on the openness scale.

There was one significant *negative* homophily effect in Study 1 (*cohort activity*: *p* < 0.001) and two in Study 2 (*conscientiousness*: *p* = 0.002; *cohort popularity*: *p* < 0.001). In Study 1, cohort members who had made few friends themselves often befriended those who had made many friends and/or vice versa; in Study 2, highly conscientious students befriended less conscientious ones, and those who were less popular befriended popular ones.

*3.4. Long term stability in friendship dynamics (H3B)*

Table 4 reports the longterm changes in friendship patterns (T2 through to T4). This represents an additional 2.5 year period from T1 in the case of Study 1, and 1.5 years from T1 in the case of Study 2. The statistical parameter is once again the odds ratio from a SIENA model.

There was no evidence for homophily further shaping the network over the longer term (i.e. after the changes in the first half year). There was, however, one significant *negative* homophily effect in Study 1 (*cohort activity*: *p* < 0.001) and three in Study 2 (*conscientiousness*: *p* = 0.024; *cohort popularity*: *p* < 0.001; *cohort activity*: *p* = 0.017), with the first two replicating the results of the short term model and the last replicating the effect in Study 1.

## 4. Discussion

Cohort members in both studies quickly formed a single interconnected community (hypothesis H1) based on a strong pattern of homophily (hypothesis H2) which showed little evidence of change in either the short term (3-6 months) or the long term (1.5-2.5 years) (hypothesis H3). Importantly, even though network density was low (~0.20), there were very few isolates: almost everyone had at least one person they counted as a friend, though a few may have been slower developing close friendships. There was strong evidence for homophily shaping the friendship patterns for a number of the investigated traits, with gender, personality, political persuasion and social engagement (e.g. popularity) being the principal factors, although with some variation in preferred traits between cohorts. As with overall cohort size, these initial homophily effects showed surprisingly little change over time. It is important to note in this context that SIENA models examine network dynamics, with the first assessment serving as the status quo from which further developments are analysed (Ripley et al., 2019). Note, therefore, that the absence of homophily effects once these had been established in the first term (a two-month period) in either the short- or longterm models does *not* imply that homophily played no role in friendship formation beyond the initial assessment. Rather, it means that that there was very little *change* in the factors influencing friendships beyond the self-assortment that had occurred in the first eight weeks.

One important feature of our study is the fact that we followed two separate, consecutive cohorts, making the conclusions we draw particularly robust. Studies 1 and 2 used slightly different ways of eliciting the number of friends and different formulations for identifying whom to include (study 1 was potentially broader than study 2). Despite these differences, the number of friends listed was almost identical in the two cohorts. The former was introduced to control for a potential recall bias, and suggests that our initial concerns were unfounded. The second was intended mainly to simplify the task for participants (at the request of the College). Again, it did not seem to have any effect as the number of names listed was identical in the two cohorts, suggesting that the general definition of ‘friend’ as someone with whom one had a meaningful relationship was widely understood (at least by British English speakers).

These findings are broadly in line with other research on student networks (Moody et al., 2011), learning networks on online courses (Fincham et al., 2018), and university student networks based on a hybrid model combining online and offline teaching (Sousa-Vieira et al., 2017). Network density was similar to that reported for other student networks (0.07-0.32: Ahn & Rodkin, 2014): this is likely to be a simple consequence of the fact that degree (the number of primary friends) is generally capped at five in both human and animal networks (Dunbar 2020, 2025; Kempnich et al. 2024). The only notable exception was with regards to network reciprocity, which seemed higher in the present case than in most previous studies, perhaps due to the unique nature of the cohort, in which students learn, live, and ‘play’ together.

Perhaps the most notable feature of these results is the speed at which stable friendships were established: within at most eight weeks of first meeting, most participants had developed friendships that lasted across the whole of their three years at university (many of which will, in fact, continue well into later life). Selfhout et al. (2010) similarly observed that the friendship formation in just-acquainted university students mainly occurred during the first three months and then tended to stabilise. This finding is in line with cellphone call data which suggest that decisions on friendship are made within 4-6 weeks of first contact (Hidd et al., 2023). The rapidity with which this happens suggests that an acute sense of threat (especially when entering a completely novel environment) prompts a hurried but focussed search for potential allies (Schachter, 1959; Stillman & Baumeister, 2009). It is possible that the small, more intimate size of colleges at the university in question (Tapper & Palfreyman, 2002) may have facilitated an especially rapid development of friendships through simple proximity.

The second finding of note is the strength of the homophily effect. The fact that this selection process happens so fast, and remains so stable after the first two months, suggests that humans are extremely good at identifying a wide range of traits in other individuals and matching these to their own. Gender and ethnicity have consistently been found to be among the strongest bases for homophily (McPherson et al., 2001). High school educational background (as a proxy for socio-economic background: Epple et al., 2004; Long & Toma, 1988) had little or no effect on friend choice in the present study, despite previous studies finding that, even when controlling for homophily in gender and ethnicity (Louch, 2000), it played a role (Skopek et al., 2011). The absence of any effects due to age (which would normally have a strong effect: Webster et al., 2018) are likely due to the fact that our participants were all very similar in age and educational achievement.

With respect to personality traits, initial friendship patterns were influenced by similarity on three of the Big Five traits (*openness*, *extraversion*, and *agreeableness*), with *conscientiousness* being identified in addition in Study 2. Beyond this initial cross-sectional assessment of homophily, Study 2 also provided evidence of homophily with regards to *openness* shaping changes in cohort network structures in the short (but not the long) run. Similar results were reported by Selfhout et al. (2010). In an earlier study of a different university cohort, Kempnich (2016) found a similar effect for these personality dimensions. Beyond these studies, however, the literature relating to personality homophily has yielded mixed findings. Some studies found personality homophily for just one of the traits (e.g., openness: Lee et al., 2009), others for two (e.g., extraversion and agreeableness: van Zalk & Denissen, 2015), and yet others for a slightly different trio (conscientiousness, agreeableness, and extraversion) (Noë et al., 2016) than that reliably observed in both of the present studies.

Under hypothesis H2, we had expected political and religious beliefs to be a significant basis for homophily (see McPherson et al. 2001). However, this was only partially supported. In both studies, left-leaning (but not right-leaning) participants nominated more same-trait friends than cohort proportions would predict. A similar effect has previously been noted in online dating, where it was estimated to be roughly half as large as an ethnicity effect (Halberstam & Knight, 2016; Huber & Malhotra, 2017). Political homophily was also observed in online environments such as twitter, again mainly for those who considered themselves left-leaning (Colleoni et al., 2014). In contrast, although previous research has found homophily effects with regards to religion (French et al., 2012), the present study did not. This might be because the majority of participants reported that they were not religious: because non-religious participants are likely to be less committed to their beliefs than religious individuals, they may be less selective, thus dampening the strength of any homophily effect on the part of religious individuals. It is possible that there was a stronger homophily effect *within* religious subgroups, though this might have shown up as ethnic homophily (see Phalet et al., 2013). Participants were not asked to indicate level of religiosity so that practising and non-practising members of a faith could not be differentiated.

Finally, there was little evidence for homophily with regards to mental health. There was some evidence that *depression* affected friendship choice in Study 1, where more same-trait friendships had been formed than expected by chance. This overall finding seemed to be driven mainly by those not diagnosed with depression selecting similar friends. Although there is some evidence of homophily in relation to depression (Hogue & Steinberg, 1995; Rosenquist et al., 2011), it may be an indirect consequence of the fact that people with depression withdraw socially, and hence are less likely to nominate, or be nominated as, friends (Chee, 2010). While homophily has been reported in respect of mental health in general (e.g., Baggio et al., 2016), there have been few attempts to investigate homophily with specific regard to anxiety. One recent study found no evidence that anxiety influenced friendship choices in a student sample (Long et al., 2020), mirroring the lack of homophily in the present sample.

The evidence that participants would tend to befriend others similar to themselves in cohort popularity and activity as well as the quantity and quality of their relationships outside the cohort (H3) was mixed. Initially, participants of both studies listed more friendships with those scoring similarly high on both cohort popularity and cohort activity than the cohort composition would predict. However, participants scoring low on both traits listed *fewer* friendships with those scoring similarly low. There is a strong suggestion from the longitudinal data that the attractiveness of high scorers on both cohort traits waned over time (Table 4). Similar findings have been noted by Dijkstra et al. (2013) and Logis et al. (2013). One possible explanation is a form of ‘status paradigm’ (Yap & Harrigan, 2015): less social individuals seek to earn social credit by associating with popular individuals – but may be less successful at sustaining these friendships because the traits they offer are less appealing to the friend.

Relationships prior to starting university affected friendship choices only to a limited extent. In Study 1, participants with a large baseline ego-network (i.e., *BEN quantity*) nominated more friends with a similar social background to themselves than cohort proportions would predict. There was, however, no evidence for *BEN quantity* shaping friendship patterns beyond this initial cross-sectional assessment in Study 1, and none whatsoever in Study 2. Furthermore, there was no evidence for *BEN quality* influencing friendship choice at any stage of the network formation process in either study. Although it has been suggested that homophily effects might stem from people’s desire to reduce uncertainty about others’ actions (Kets & Sandroni, 2019), the present results provide only limited support for such a suggestion.

One explanation for some of the results in Study 1 might be that participants were shown all their previously listed friends and therefore had to make a conscious decision to drop them: the threshold for making a choice might hence be higher than that for simply forgetting to list a friend from a previous wave (Bell et al., 2007; Neyer, 1997). As a result, networks might have appeared more static (and hence larger) than they actually were. Indeed, the Jaccard indices for Study 1 indicate that 87% and 89% of network ties remained static between successive assessments in the first year. The recognition-based nomination procedure used in Study 2 certainly produced more dynamic networks (Jaccard indices of only 47%), but the two studies’ results were extremely similar, suggesting that the nomination procedure probably had a limited effect. More importantly, the SIENA model rate parameters indicate that there was more than sufficient network change between assessments in Study 1 to allow meaningful modelling, making the explanation less plausible in this case.

Although the complete network approach we used avoids most of the limitations that characterise many network studies (Wölfer et al., 2015), self-assessments of social traits are notoriously susceptible to bias effects. Differentiating between perceived, actual, and peer-rated similarity can yield diverging results in terms of personality homophily (Selfhout et al., 2009). More objective measures for assessing traits would be desirable. Studies deriving personality characteristics from social media data (Azucar et al., 2018; Golbeck et al., 2011; Park et al., 2015) suggest one promising avenue for future research, especially if combined with self-rated data. Youyou et al. (2017), for instance, confirmed personality homophily in friendships using such a blended approach.

One could argue that, in the rapidly evolving era of the internet, it is no longer necessary to share a physical space for relationships to develop, and hence that the present results might not be representative of actual experience in the contemporary world. However, ‘offline’ physical proximity plays a major part in determining who connects in virtual space (Huang et al., 2013). In fact, there is considerable evidence to suggest that face-to-face communication remains both the dominant mode (Baym et al., 2004) and the preferred mode (Vlahovic et al. 2012) of interaction for most university students.

*Future Directions*

This study raises a number of issues that would merit more detailed exploration. One important one would be to study a wider variety of contexts that simply adolescents. Do older adults exhibit the same patterns of friendship replacement on moving for work or retirement, and at the same breakneck speed? It may be helpful, also, to explore a wider range of traits as the basis for homophily. Different surface and deep forms of homophily trait may well perform differently in different contexts and for different ages of indiuviduals. We did not find striking gender differences in our dataset, even though we know gender introduces very striking differences in network size and structure (Dunbar et al., 2024). We know little about homophily preferences in relation to gender.

The present results also have implications for any context in which a random group of strangers is brought together, such as a work environment, the military, or schools. Since friendships form very quickly, it may be important for group efficiency and effectiveness to provide time and opportunity for individuals to mix socially so that a sense of belonging develops quickly. Segal (1974), for example, found that merely seating people next to one another (determined by something as simple as alphabetical order) can result in long-lasting friendships. Similarly, assigning individuals to specific subgroups during an induction event lasting a mere few hours can create more diverse friendships in a cohort (Boda et al., 2020). This might have significant implications for integration in racially heterogenous schools (Wells et al. 2016). Although we would caution against one-shot solutions (meaningful friendships *do* take time to develop: Hall 2012), employing strategies like these would pay dividends in terms of employee engagement and functional effectiveness (see also Camilleri et al. 2023). More detailed exploration of the possibilities would surely repay investment of research effort.

**Acknowledgments**

The study was funded by a UK Medical Research Council graduate studentship to MK and a European Research Council Advanced grant (RELNET) and an EU Horizon 2020 research grant (IBSEN) to RD. We thank the Governing Body of the college for permission to conduct the project.

**Declaration of Interests:** none.

**Ethics Statement:** The studies reported herein were carried out in accordance with the ethical standards of the responsible committee on human experimentation (institutional and national) and with the Helsinki Declaration of 1975 (as revised in 2008 (5)).

**Disclosure:** At no stage in the preparation of this manuscript was any form of AI or AI-assisted technologies.
**References**

Ahn, H. & Rodkin, P. (2014). Classroom-level predictors of the social status of aggression: Friendship centralization, friendship density, teacher–student attunement, and gender. *Journal of Educational Psychology* 106: 1144–1155.

Azucar, D., Marengo, D. & Settanni, D. (2018). Predicting the Big 5 personality traits from digital footprints on social media: A meta-analysis. *Personality and Individual Differences* 124: 150–159.

Baggio, S., Luisier, V. & Vladescu, C. (2016). Relationships between social networks and mental health. *Swiss Journal of Psychology* 76: 5–11.

Baldasaro, R. E., Shanahan, M. J., & Bauer, D. J. (2013). Psychometric properties of the mini-IPIP in a large, nationally representative sample of young adults. *Journal of Personality Assessment* 95: 74–84.

Baumeister, R. F., & Leary, M. R. (1995). The need to belong: Desire for interpersonal attachments as a fundamental human motivation. *Psychological Bulletin* 117: 497–529.

Baym, N. K., Zhang, Y. B., & Lin, M. C. (2004). Social interactions across media: Interpersonal communication on the internet, telephone and face-to-face. *New Media & Society* 6: 299–318.

Bell, D., Belli-McQueen, B., & Haider, A. (2007). Partner naming and forgetting: recall of network members. *Social Networks* 29: 279–299.

Berg, J. H., & Clark, M. S. (1986). Differences in social exchange between intimate and other relationships: Gradually evolving or quickly apparent? In *Friendship and Social Interaction* (pp. 101–128). Springer.

Blieszner, R., & Adams, R. G. (1992). *Adult Friendship*. Sage Publications.

Block, P., & Grund, T. (2014). Multidimensional homophily in friendship networks. *Network Science* 2: 189–212.

Blumenstock, J., Toomet, O., Ahas, R., & Saluveer, E. (2015). Neighborhood and network segregation: ethnic homophily in a “silently separate society.” *Proceedings of NetMob 2015*. https://www.cerge.cuni.cz/pdf/gdn/rrc/RRC13_50_paper_01.pdf

Boda, Z., Elmer, T., Vörös, A., & Stadtfeld, C. (2020). Short-term and long-term effects of a social network intervention on friendships among university students. *Scientific Reports* 10: 2889.

van de Bunt, G. G., J Van Duijn, M. A., Snijders, T. (1999). Friendship networks through time: An actor-oriented dynamic statistical network model. *Computational & Mathematical Organization Theory*, *5*(2), 167–192. https://doi.org/10.1023/A:1009683123448

Burt, R. S. (1987). A note on missing network data in the general social survey. *Social Networks* 9: 63–73.

Cacioppo, J. T. & Patrick, W. (2008). *Loneliness: Human Nature and the Need for Social Connection*. New York: Norton.

Cacioppo, J. T., Cacioppo, S. & Boomsma, D. I. (2014). Evolutionary mechanisms for loneliness. *Cognition & Emotion* 28: 3-21.

Cacioppo, J. T., Hawkley, L. C., Crawford, L. E., Ernst, J. M., Burleson, M. H., Kowalewski, R.B., ... & Berntson, G.G. (2002). Loneliness and health: Potential mechanisms. *Biopsychosocial Science and Medicine* 64: 407-417.

Cameron, E.Z., Setsaas, T. H. & Linklater, W. L. (2009). Social bonds between unrelated females increase reproductive success in feral horses. *Proceedings of the National Academy of Sciences USA* 106: 13850-13853.

Camilleri, T., Rocky, S. & Dunbar, R.I.M. (2023). *The Social Brain: The Psychology of Successful Groups.* London: Penguin.

Chee, B. (2010). Sickness and health: Homophily in online health forums. *iConference*. https://www.ideals.illinois.edu/items/14899

Colleoni, E., Rozza, A., & Arvidsson, A. (2014). Echo chamber or public sphere? Predicting political orientation and measuring political homophily in twitter using big data. *Journal of Communication* 64: 317-332.

Curry, O. & Dunbar, R.I.M. (2011). Altruism in networks: the effect of connections. *Biological Letters* 7: 651-653.

Curry, O. & Dunbar, R.I.M. (2013). Do birds of a feather flock together? The relationship between similarity and altruism in social networks. *Human Nature* 24: 336-347*.*

Dijkstra, J. K., Cillessen, A. H. N., & Borch, C. (2013). Popularity and adolescent friendship networks: Selection and influence dynamics. *Developmental Psychology* 49: 1242–1252.

Donnellan, M. B., Oswald, F. L., Baird, B. M., & Lucas, R. E. (2006). The mini-IPIP scales: Tiny-yet-effective measures of the Big Five factors of personality. *Psychological Assessment* 18: 192–203. <https://doi.org/10.1037/1040-3590.18.2.192>

Dunbar, R.I.M. (2018). The anatomy of friendship. *Trends in Cognitive Sciences* 22: 32-51.

Dunbar, R.I.M. (2020). Structure and function in human and primate social networks: Implications for diffusion, network stability and health. *Proceedings of the Royal Society, London,* 476A: 20200446.

Dunbar, R.I.M. (2025a). Structural and cognitive mechanisms of group cohesion in primates. *Behavioral and Brain Sciences* 48: e162.

Dunbar, R.I.M. (2025b). Why friendship and loneliness affect our health. *Annals of the New York Academy of Sciences* 1545: 52-65.

Dunbar, R.I.M., Pearce, E., Wlodarski, R. & Machin, A. (2024). Sex differences in close friendships and social style: an evolutionary perspective. *Evolution and Human Behavior* 45: 106631.

Eisenberg, D., Gollust, S. E., Golberstein, E., & Hefner, J. L. (2007). Prevalence and correlates of depression, anxiety, and suicidality among university students. *American Journal of Orthopsychiatry* 77: 534–542. https://doi.org/10.1037/0002-9432.77.4.534

Ekstrom, R. B. (1961). Experimental studies of homogeneous grouping: A critical review. *The School Review* 69: 216-226. https://doi.org/10.1086/442582

Epple, D., Figlio, D., & Romano, R. (2004). Competition between private and public schools: Testing stratification and pricing predictions. *Journal of Public Economics* 88: 1215-1245.

Escribano, D., Lapuente, F., Cuesta, J., Dunbar, R.I.M. & Sánchez, A. (2023). Longitudinal study of friendships and enmities in middle school. *Scientific Reports* 13: 14575.

Farley, R., Steeh, C., Krysan, M., Jackson, T., & Reeves, K. (1994). Stereotypes and segregation: Neighborhoods in the Detroit area. *American Journal of Sociology* 100: 750–780. https://doi.org/10.1086/230580

Feld, S. L., & Carter, W. C. (2002). Detecting measurement bias in respondent reports of personal networks. *Social Networks* 24: 365–383.

Felton, B. J., & Shinn, M. (1992). Social integration and social support: Moving “social support” beyond the individual level. *Journal of Community Psychology* 20: 103–115. https://doi.org/10.1002/1520-6629(199204)20:2<103::AID-JCOP2290200202>3.0.CO;2-4

Festinger, L. (1954). A theory of social comparison processes. *Human Relations* 7: 117–140. https://doi.org/10.1177/001872675400700202

Fincham, E., Gašević, D., & Pardo, A. (2018). From social ties to network processes: Do tie definitions matter? *Journal of Learning Analytics* 5: 9–28.

French, D. C., Purwono, U., & Rodkin, P. C. (2012). Religiosity of adolescents and their friends and network associates: Homophily and associations with antisocial behavior. *Journal of Research on Adolescence* 22: 326–332. https://doi.org/10.1111/j.1532-7795.2012.00778.x

Gere, J., & Macdonald, G. (2010). An update of the empirical case for the need to belong. *Journal of Individual Psychology* 66: 93–115.

Godley, J. (2008). Preference or propinquity? The relative contribution of selection and opportunity to friendship homophily in college. *Connections* 1: 65–80.

Golbeck, J., Robles, C., & Turner, K. (2011). Predicting personality with social media. *Conference on Human Factors in Computing Systems – Proceedings*, pp. 253–262. https://doi.org/10.1145/1979742.1979614

Goldberg, L. R. (1999). A broad-bandwidth, public domain, personality inventory measuring the lower-level facets of several five-factor models. *Personality Psychology in Europe* 7: 7–28.

Graham, J. A., Cohen, R., Zbikowski, S. M., & Secrist, M. E. (1998). A longitudinal investigation of race and sex as factors in children’s classroom friendship choices. *Child Study* 28: 245–266.

Hafen, C. A., Laursen, B., Burk, W. J., Kerr, M., & Stattin, H. (2011). Homophily in stable and unstable adolescent friendships: Similarity breeds constancy. *Personality and Individual Differences* 51: 607–612. https://doi.org/10.1016/j.paid.2011.05.027

Halberstam, Y., & Knight, B. (2016). Homophily, group size, and the diffusion of political information in social networks: Evidence from Twitter. *Journal of Public Economics* 143: 73–88. <https://doi.org/10.1016/j.jpubeco.2016.08.011>

Hall, J.A. (2012). Friendship standards: the dimensions of ideal expectations. *Journal of Social and Personal Relationships* 29: 884-907.

Hays, R. B. (1985). A longitudinal study of friendship development. *Journal of Personality and Social Psychology* 48: 909–924.

Hechanova-Alampay, G., Beehr, T. A., Christiansen, N. D., & Van Horn, R. K. (2002). Adjustment and strain among domestic and international student sojourners: A longitudinal study. *School Psychology International* 23: 458-474. <https://doi.org/10.1177/0143034302234007>

Hidd, V., López, E., Centellegher, S., Roberts, S.B.G., Lepri, B. & Dunbar, R.I.M. (2023). The stability of transient relationships. *Scientific Reports* 13: 6120.

Hogue, A., & Steinberg, L. (1995). Homophily of internalized distress in adolescent peer groups. *Developmental Psychology* 31: 897–906.

Huang, G. C., Soto, D., Fujimoto, K., & Valente, T. W. (2014). The interplay of friendship networks and social networking sites: Longitudinal analysis of selection and influence effects on adolescent smoking and alcohol use. *American Journal of Public Health* 104: e51-e59. https://doi.org/10.2105/AJPH.2014.302038

Huang, Y., Shen, C., & Contractor, N. S. (2013). Distance matters: Exploring proximity and homophily in virtual world networks. *Decision Support Systems* 55: 969–977.

Huber, G. A., & Malhotra, N. (2017). Political homophily in social relationships: Evidence from online dating behavior. *Journal of Politics* 79: 269–283. https://doi.org/10.1086/687533

Huisman, M. (2014). Imputation of missing network data: Some simple procedures. In R. Alhajj & J. Rokne (Eds.), *Encyclopedia of Social Network Analysis and Mining* (pp. 708–742). Springer.

Huisman, M., & Steglich, C. (2008). Treatment of non-response in longitudinal network studies. *Social Networks* 30: 297–308. https://doi.org/10.1016/j.socnet.2008.04.004

Hwang, E. H., Singh, P. V., & Argote, L. (2012). Learning to cross boundaries in online knowledge communities: Fading of surface-level and rise of deep-level similarity with experience. *Carnegie Mellon University Tepper School of Business Working Paper.*

Kandel, D. B., Davies, M., & Baydar, N. (1990). The creation of interpersonal contexts: Homophily in dyadic relationships in adolescence and young adulthood. In L. N. Robins & M. Rutter (Eds.), *Straight and devious pathways for childhood to adulthood* (pp. 221–241). Cambridge University Press.

Kempnich, M. (2016). *To befriend or not to befriend – A longitudinal study showing the influence of propinquity on friendship choice diminishes as that of homophily grows*. MSc thesis, University of Oxford.

Kempnich, M., Wölfer, R., Hewstone, M. & Dunbar, R.I.M. (2024). How the size and structure of egocentric networks change during a life transition. *Advances in Life Course Research* 61: 100632.

Kessler, R. C., Berglund, P., Demler, O., Jin, R., Koretz, D., Merikangas, K., & Wang, P. (2003). The epidemiology of major depressive disorder: results from the National Comorbidity Survey Replication (NCS-R). *Jama: The Journal of the American Medical Association* 289: 3095–3105.

Kessler, R. C., & Walters, E. E. (1998). Epidemiology of DSM-III-R major depression and minor depression among adolescents and young adults in the National Comorbidity Survey. *Depression and Anxiety* 7: 3–14. https://doi.org/10.1002/(SICI)1520-6394(1998)7:1<3::AID-DA2>3.0.CO;2-F

Kets, W., & Sandroni, A. (2019). A belief-based theory of homophily. *Games and Economic Behavior* 115: 410–435.

King, D. D., Ott-Holland, C. J., Ryan, A. M., Huang, J. L., Wadlington, P. L., & Elizondo, F. (2017). Personality homogeneity in organizations and occupations: Considering similarity sources. *Journal of Business and Psychology* 32: 641–653. https://doi.org/10.1007/s10869-016-9459-4

Kroenke, K., & Spitzer, R. L. (2002). The PHQ-9: a new depression diagnostic and severity measure. *Psychiatric Annals* 32: 509–515.

Laursen, B., Popp, D., Burk, W., Kerr, M., & Stattin, H. (2011). Incorporating interdependence into developmental research: Examples from the study of homophily and homogeneity. In N. A. Card, J. P. Selig, & T. Little (Eds.), *Modeling dyadic and interdependent data in the developmental and behavioural sciences* (pp. 21–48). Routledge.

Lazarsfeld, P. F., & Merton, R. K. (1954). Friendship as a social process: A substantive and methodological analysis. In M. Berger, T. Abel, & C. H. Page (Eds.), *Freedom and control in modern society* (pp. 18–66). Octagon Books.

Lee, K., Ashton, M. C., & Visser, B. (2009). Similarity and assumed similarity in personality reports of well-acquainted persons. *Journal of Personality and Social Psychology* 96: 460–472. https://doi.org/10.1037/a0014059

Logis, H. A., Rodkin, P. C., Gest, S. D., & Ahn, H. J. (2013). Popularity as an organizing factor of preadolescent friendship networks: Beyond prosocial and aggressive behavior. *Journal of Research on Adolescence* 23: 413–423. https://doi.org/10.1111/jora.12033

Long, E., Gardani, M., McCann, M., Sweeting, H., Tranmer, M., & Moore, L. (2020). Mental health disorders and adolescent peer relationships. *Social Science & Medicine* 253: 112973.

Long, J. E. & Toma, E. F. (1988). The determinants of private school attendance, 1970-1980. *Review of Economics and Statistics* 70: 351-357.

Louch, H. (2000). Personal network integration: Transitivity and homophily in strong-tie relations. *Social Networks* 22: 45–64.

Marsden, P. V. (1988). Homogeneity in confiding relations. *Social Networks* 10: 57–76.

Marsden, P. V. (1990). Network data and measurement. *Annual Review of Sociology* 16: 435–463. https://doi.org/10.1146/annurev.so.16.080190.002251

Maslow, A. H. (1943). A theory of human motivation. *Psychological Review* 50: 370–396.

McCrae, R., & Costa, P. (2003). *Personality in adulthood: A five-factor theory perspective*. Guilford Press.

McPherson, M., Smith-Lovin, L., & Cook, J. M. (2001). Birds of a feather: Homophily in social networks. *Annual Review of Sociology* 27: 415–444.

Mercken, L., Steglich, C., Sinclair, P., & Holliday, J. (2012). A longitudinal social network analysis of peer influence, peer selection and smoking behaviour in British schools. *Health Psychology* 31: 450–459. https://doi.org/10.1037/a0026876

Moody, J., Brynildsen, W., Osgood, D., & Feinberg, M. (2011). Popularity trajectories and substance use in early adolescence. *Social Networks* 33: 101–112.

Neyer, F. J. (1997). Free recall or recognition in collecting egocentered networks: The role of survey techniques. *Journal of Social and Personal Relationships* 14: 305–316. https://doi.org/10.1177/0265407597143002

Noë, N., Whitaker, R. M., & Allen, S. M. (2016). Personality homophily and the local network characteristics of Facebook. *Proceedings of the 2016 IEEE/ACM International Conference on Advances in Social Networks Analysis and Mining*, pp. 386–393.

Nuñez, C.M.V. et al. (2015). Sociality increases juvenile survival after a catastrophic event in the feral horse (*Equus caballus*). *Behavioral Ecology* 26: 138-147

Orne, M. T. (1962). On the social psychology of the psychological experiment: With particular reference to demand characteristics and their implications. *American Psychologist* 17: 776–783.

Park, G., Schwartz, H. A., Eichstaedt, J. C., Kern, M. L., Kosinski, M., Stillwell, D. J., Ungar, L. H., & Seligman, M. E. (2015). Automatic personality assessment through social media language. *Journal of Personality and Social Psychology*,108: 934–948. https://doi.org/10.1037/pspp0000020

Phalet, K., Leuven, K. U., & Maliepaard, M. (2013). The making and unmaking of religious boundaries: Comparing Turkish and Moroccan Muslim minorities in European cities. *Comparative Migration Studies* 1: 123–145. https://doi.org/10.5117/CMS2013.1.PHAL

Phillips, D. L. (2006). Parallel lives? Challenging discourses of British Mus:lim self-segregation. *Environment and Planning D: Society and Space* 24 25–40. https://doi.org/10.1068/d60j

Phillips, D. L., & Clancy, K. J. (1972). Some effects of “social desirability” in survey studies. *American Journal of Sociology* 77: 921–940. https://doi.org/10.1086/225231

Reagans, R. (2011). Close encounters: Analyzing how social similarity and propinquity contribute to strong network connections. *Organization Science* 22: 835–849. https://doi.org/10.1287/orsc.1100.0587

Ripley, R. M., Snijders, T. A. B., Boda, Z., Vörös, A., & Preciado, P. (2019). *Manual for RSiena version 4.0 (version May 21, 2019)*. University of Oxford, Department of Statistics; Nuffield College.

Roberts, S.B.G. & Dunbar, R.I.M. (2011). The costs of family and friends: an 18-month longitudinal study of relationship maintenance and decay. *Evolution and Human Behavior* 32: 186-197.

Roberts, S.B.G., Arrow, H., Lehmann, J. & Dunbar, R.I.M. (2014). Close social relationships: an evolutionary perspective. In: R.I.M. Dunbar, C. Gamble & J.A.J. Gowlett (eds) *Lucy to Language: the Benchmark Papers*, pp. 151-180. Oxford: Oxford University Press.

Rosenquist, J., Fowler, J., & Christakis, N. (2011). Social network determinants of depression. *Molecular Psychiatry* 16: 273-281.

Roy, C., Bhattacharya, K., Dunbar, R. & Kaski, K. (2022). Turnover in close friendships. *Scientific Reports* 12: 11018.

Saramäki, J., Leicht, E., López, E., Roberts, S., Reed-Tsochas, F. & Dunbar, R.I.M. (2013). The persistence of social signatures in human communication. *Proceedings of the National Academy of Sciences USA* 111: 942-947.

Schachter, S. (1959). *The Psychology of Affiliation: Experimental Studies of the Sources of Gregariousness.* Stanford University Press.

Schaefer, D. R., Kornienko, O., & Fox, A. M. (2011). Misery does not love company: Network selection mechanisms and depression homophily. *American Sociological Review* 76: 764–785. https://doi.org/10.1177/0003122411420813

Scull, S., & Cuthill, M. (2010). Engaged outreach: Using community engagement to facilitate access to higher education for people from low socio-economic backgrounds. *Higher Education Research & Development* 29: 59–74. https://doi.org/10.1080/07294360903421368

Segal, M. W. (1974). Alphabet and attraction: An unobtrusive measure of the effect of propinquity in a field setting. *Journal of Personality and SocialPsychology* 30: 654–657.

Selfhout, M., Burk, W., Branje, S., Denissen, J., van Aken, M., & Meeus, W. (2010). Emerging late adolescent friendship networks and big five personality traits: A social network approach. *Journal of Personality* 78: 509–538. https://doi.org/10.1111/j.1467-6494.2010.00625.x

Selfhout, M., Denissen, J., Branje, S., & Meeus, W. (2009). In the eye of the beholder: Perceived, actual, and peer-rated similarity in personality, communication, and friendship intensity during the acquaintanceship process. *Journal of Personality and Social Psychology* 96: 1152–1165. https://doi.org/10.1037/a0014468

Shavit, Y., Arum, R., & Gamoran, A. (2007). *Stratification in Higher Education: A Comparative Study*. Stanford University Press.

Shin, H., & Ryan, A. M. (2014). Early adolescent friendships and academic adjustment: Examining selection and influence processes with longitudinal social network analysis. *Developmental Psychology* 50: 2462–2472.

Silk, J.B., Beehner, J.C., Bergman, T., Crockford, C., et al. (2009). The benefits of social capital: close social bonds among female baboons enhance offspring survival. *Proceedings of the Royal Society, London,* 276: 3099-3104.

Silk, J.B., Beehner, J.C., Bergman, T., Crockford, C., et al. (2010). Strong and consistent social bonds enhance the longevity of female baboons. *Current Biology* 20: 1359-1361.

Skopek, J., Schulz, F., & Blossfeld, H. (2011). Who contacts whom? Educational homophily in online mate selection. *European Sociological* 27: 180–195.

Snijders, T. A. B., Van De Bunt, G. G., & Steglich, C. E. G. (2010). Introduction to stochastic actor-based models for network dynamics. *Social Networks* 32: 44–60. https://doi.org/10.1016/j.socnet.2009.02.004

Sousa-Vieira, M. E., López-Ardao, J. C., & Fernández-Veiga, M. (2017). Characterizing social interactions in online social networks: The case of university students. *International Conference on Computer Supported Education* 2: 188–199. https://doi.org/10.5220/0006292701880199

Spitzer, R., Kroenke, K., Williams, J. B., & Löwe, B. (2006). A brief measure for assessing generalized anxiety disorder: the GAD-7. *Archives of Internal Medicine* 166: 1092–1097.

Steglich, C., Snijders, T. A. B., & West, P. (2006). Applying SIENA: An illustrative analysis of the coevolution of adolescents’ friendship networks, taste in music, and alcohol consumption. *Methodology* 2: 48–56. https://doi.org/10.1027/1614-2241.2.1.48

Stillman, T. F., & Baumeister, R. F. (2009). Uncertainty, belongingness, and four needs for meaning. *Psychological Inquiry* 20: 249–251.

https://doi.org/10.1080/10478400903333544

Tapper, T., & Palfreyman, D. (2002). Understanding collegiality: The changing Oxbridge model. *Tertiary Education and Management* 8: 47–63. https://doi.org/10.1023/A:1017967104176

Taylor, S. E. (2006). Tend and befriend: Biobehavioral bases of affiliation under stress. *Current Directions in Psychological Science* 15: 273–277. <https://doi.org/10.1111/j.1467-8721.2006.00451.x>

Tinbergen, N. (1963). On aims and methods of ethology. *Zeitschrift für Tierpsychologie*

20; 410-433

Tran, L. T. (2016). Students’ academic, intercultural and personal development in globalised education mobility. In C. Ng, R. Fox, & M. Nakano (Eds.), *Reforming learning and teaching in Asia-Pacific universities* (Vol. 33, pp. 95–113). Springer. https://doi.org/10.1007/978-981-10-0431-5_5

Veenstra, R., Kornelis Dijkstra, J., Steglich, C., W Van Zalk, M. H., Munniksma, A., Rambaran, A., Oldenburg, B., Ruschoff, B., Duijst, D., Munniksma, F., Huitsing, G., Sijtsema, J., Al, J. P., Pattiselanno, K., van Rijsewijk, L., Firnenburg, L., Sentse, M., van der Ploeg, R., Kretschmer, T., … Heininga, V. (2013). Network-Behavior Dynamics. *Journal of Research on Adolescence* 23: 399–412. https://doi.org/10.1111/jora.12070

Verbrugge, L. M. (1977). The structure of adult friendship choices. *Social Forces* 56: 576–597.

Vlahovic, T., Roberts, S.B.G. & Dunbar, R.I.M. (2012) Effects of duration and laughter on subjective happiness within different modes of communication. *Journal of Computer-Mediated Communication* 17: 436-450.

Webster, N., Antonucci, T. & Ajrouch, K. (2018). Educational homophily within social networks: Cohort effects over time. *Innovation in Aging* 2: 37.

Weerman, F. M. (2011). Delinquent peers In context: a longitudinal network analysis of selection and influence effects. *Criminology* 49: 253-286. https://doi.org/10.1111/j.1745-9125.2010.00223.x

Weitzman, E. R. (2004). Poor mental health, depression, and associations with alcohol consumption, harm, and abuse in a national sample of young adults in college. *Journal of Nervous and Mental Disease* 192: 269-277.

Wells, A., Fox, L., & Cordova-Cobo, D. (2016). How racially diverse schools and classrooms can benefit all students. *Education Digest* 82: 17-24.

Wittig, R.M., Crockford, C., Lehmann, J., et al. (2008). Focused grooming networks and stress alleviation in wild female baboons. *Hormones and Behavior* 54: 170-177.

Wölfer, R., Faber, N. S., & Hewstone, M. (2015). Social network analysis in the science of groups: Cross-Sectional and longitudinal applications for studying intra-and intergroup behavior. *Group Dynamics: Theory, Research, and Practice* 19: 45–61. https://doi.org/10.1037/gdn0000021

Yap, J., & Harrigan, N. (2015). Why does everybody hate me? Balance, status, and homophily: The triumvirate of signed tie formation. *Social Networks* 40: 103–122.

Youyou, W., Stillwell, D., Schwartz, H. A., & Kosinski, M. (2017). Birds of a feather do flock together: Behavior-based personality-assessment method reveals personality similarity among couples and friends. *Psychological Science* 28: 276–284. https://doi.org/10.1177/0956797616678187

Zandberg, T., & Huisman, M. (2019). Missing behavior data in longitudinal network studies: The impact of treatment methods on estimated effect parameters in stochastic actor oriented models. *Social Network Analysis and Mining* 9: 8. https://doi.org/10.1007/s13278-019-0553-2

Zeggelink, E. (1993). *Strangers Into Friends: The Evolution of Friendship Networks Using an Individual Oriented Modeling Approach*. PhD thesis, University of Groningen.

van Zalk, M., & Denissen, J. (2015). Idiosyncratic versus social consensus approaches to personality: Self-view, perceived, and peer-view similarity. *Journal of Personality and Social Psychology* 109: 121–141.

Zipf, G. (1949). *Human Behavior and the Principle of Least Effort.* Addison-Wesley.

Table 1. Initial homophily in Study 1 after one term

| **Source of similarity** | **OVERALL**  **proportion of**  **same-trait friends (SD)** |  | **SUBGROUP**  **proportion of**  **same-trait**  **friends (SD)** | **Trait distribution in cohort (proportion)** |
| --- | --- | --- | --- | --- |
| Gender | 0.53 (0.16) | < | 0.55 (0.15) | Male (0.56) |
|  |  |  | **0.50 (0.17)***** | Female (0.44) |
| Ethnicity | **0.66 (0.27)******* | *<* | 0.79 (0.11) | White (0.73) |
|  |  |  | 0.25 (0.22) | Non-white (0.27) |
| School background | 0.52 (0.17) | < | 0.57(0.18) | State (0.54) |
|  |  |  | 0.46 (0.14) | Private (0.46) |
| Openness | **0.60 (0.22)****** | < | 0.70 (0.15) | High (0.69) |
|  |  |  | 0.34 (0.15) | Low (0.31) |
| Conscientiousness | 0.50 (0.18) | < | 0.49 (0.19) | High (0.51) |
|  |  |  | 0.51 (0.18) | Low (0.49) |
| Extraversion | **0.55 (0.20)*** | *<* | **0.65 (0.18)******* | High (0.51) |
|  |  |  | 0.46 (0.16) | Low (0.49) |
| Agreeableness | 0.52 (0.16) | *<* | **0.57 (0.15)****** | High (0.51) |
|  |  |  | 0.46 (0.14) | Low (0.49) |
| Neuroticism | 0.50 (0.16) | < | 0.51 (0.17) | High (0.51) |
|  |  |  | 0.50 (0.16) | Low (0.49) |
| Political identity | 0.53 (0.23) | *<* | **0.63 (0.19)****** | Left (0.54) |
|  |  |  | 0.39 (0.22) | Right (0.46) |
| Religion | 0.49 (0.16) | < | 0.56 (0.15) | Non-religious (0.56) |
|  |  |  | 0.40 (0.13) | Religious (0.44) |
| Anxiety | 0.51 (0.16) | < | 0.49 (0.16) | Yes (0.44) |
|  |  |  | 0.51 (0.18) | No (0.56) |
| Depression | **0.54 (0.17)***** | < | 0.46 (0.18) | Yes (0.46) |
|  |  |  | **0.59 (0.16)***** | No (0.54) |
| Cohort popularity | 0.55 (0.27) | < | **0.75 (0.16)******* | High (0.51) |
|  |  |  | **0.32 (0.16)******* | Low (0.49) |
| Cohort activity | 0.51 (0.32) | < | **0.71 (0.10)******* | High (0.51) |
|  |  |  | **0.24 (0.32)******* | Low (0.49) |
| BEN quantity† | 0.53 (0.18) | < | 0.56 (0.15)* | High (0.51) |
|  |  |  | 0.49 (0.21) | Low (0.49) |
| BEN quality† | 0.51 (0.15) | < | 0.52 (0.15) | High (0.51) |
|  |  |  | 0.50 (0.15) | Low (0.49) |

Significant cells are bolded: * *p*≤0.05	** *p*≤0.001	*** *p*≤0.001

< specifies a significant difference between subgroups (p≤0.05)

† BEN quantity: number of home friends listed at T0 [immediately before coming to university]; BEN quality: emotional closeness to these individual home friends, rated on a 1 [neutral]-10 [very close] analogue scale.

Table 2. Initial homophily in Study 2 after one term

| **Source of similarity** | **OVERALL**  **proportion of**  **same-trait**  **friends (SD)** |  | **SUBGROUP**  **proportion of**  **same-trait**  **friends (SD)** | **Trait distribution in cohort (proportion)** |
| --- | --- | --- | --- | --- |
| Gender | **0.56 (0.14)******* | < | 0.52 (0.17) | Male (0.51) |
|  |  |  | **0.60 (0.11)****** | Female *(0.49)* |
| Ethnicity | **0.61 (0.22***)**** | *<* | 0.72 (0.11) | White (0.74) |
|  |  |  | 0.29 (0.09) | Non-white (0.26) |
| School background | 0.51 (0.15) | < | 0.56(0.14) | State (0.56) |
|  |  |  | 0.44 (0.13) | Private (0.44) |
| Openness | **0.54 (0.17)***** | < | 0.62 (0.15) | High (0.59) |
|  |  |  | 0.42 (0.12) | Low (0.41) |
| Conscientiousness | 0.53 (0.17) | < | **0.58 (0.15)****** | High *(0.64)* |
|  |  |  | **0.43 (0.09)***** | Low (0.36) |
| Extraversion | **0.53 (0.15)***** | *<* | **0.72 (0.10)******* | High *(0.60)* |
|  |  |  | 0.38 (0.17) | Low (0.40) |
| Agreeableness | **0.57 (0.21)****** | *<* | 0.55 (0.14) | High (0.52) |
|  |  |  | 0.51 (0.14) | Low (0.48) |
| Neuroticism | 0.49 (0.13) | < | 0.64 (0.12) | High (0.63) |
|  |  |  | 0.37 (0.11) | Low (0.37) |
| Political identity | **0.57 (0.21)****** | *<* | **0.70 (0.11)***** | Left (0.62) |
|  |  |  | 0.35 (0.15) | Right (0.38) |
| Religion | 0.50 (0.15) | < | 0.56 (0.12) | Non-religious (0.58) |
|  |  |  | 0.42 (.15) | Religious (0.42) |
| Anxiety | 0.48 (0.15) | < | 0.33 (0.15) | Yes (0.33) |
|  |  |  | 0.65 (0.16) | No (0.67) |
| Depression | 0.49 (0.15) | < | 0.40 (0.17) | Yes (0.38) |
|  |  |  | 0.58 (0.13) | No (0.62) |
| Cohort popularity | 0.53 (0.29) | < | **0.79 (0.07)******* | High (0.51) |
|  |  |  | **0.26 (0.16)******* | Low (0.49) |
| Cohort activity | 0.51 (0.20) | < | **0.64 (0.06***)**** | High (0.53) |
|  |  |  | **0.37 (0.20)****** | Low (0.47) |
| BEN quality | 0.50 (0.16) | < | 0.53 (0.14) | High (0.51) |
|  |  |  | 0.47 (0.14) | Low (0.49) |
| BEN quantity | 0.50 (0.14) | < | 0.46 (0.18) | High (0.51) |
|  |  |  | 0.53 (0.14) | Low (0.49) |

Significant cells are **bolded**: * *p*≤0.05	** *p*≤0.001	*** *p*≤0.001

< specifies a significant difference between subgroups (p≤0.05)

Table 3. SIENA estimation results for the short-term models for both studies, with network size as the outcome variable.

| Trait | Study 1 | | | Study 2 | | |
| --- | --- | --- | --- | --- | --- | --- |
|  | **Odds ratio** | **SE** | ***p*** | **Odds ratio** | **SE** | ***p*** |
| Structural Controls |  |  |  |  |  |  |
| outdegree (density) | **-3.37** | **0.27** | **<0.001** | **-3.86** | **0.29** | **<0.001** |
| reciprocity | **1.47** | **0.14** | **<0.001** | **1.29** | **0.10** | **<0.001** |
| transitive triplets | **0.21** | **0.02** | **<0.001** | **0.18** | **0.01** | **<0.001** |
| transitive ties | **0.89** | **0.21** | **<0.001** | **1.03** | **0.30** | **<0.001** |
| Gender |  |  |  |  |  |  |
| homophily | **0.30** | **0.11** | **0.008** | 0.06 | 0.07 | 0.391 |
| alter | **-0.47** | **0.14** | **0.001** | 0.07 | 0.09 | 0.465 |
| ego | 0.59 | 0.22 | 0.008 | 0.11 | 0.09 | 0.244 |
| Ethnicity |  |  |  |  |  |  |
| homophily | -0.10 | 0.15 | 0.503 | 0.07 | 0.09 | 0.459 |
| alter | 0.08 | 0.16 | 0.622 | -0.23 | 0.12 | 0.056 |
| ego | **0.42** | **0.20** | **0.043** | -0.04 | 0.12 | 0.740 |
| School background |  |  |  |  |  |  |
| homophily | 0.15 | 0.11 | 0.175 | 0.07 | 0.07 | 0.305 |
| alter | -0.08 | 0.12 | 0.515 | 0.04 | 0.09 | 0.662 |
| ego | **0.71** | **0.22** | **0.001** | 0.12 | 0.09 | 0.175 |
| Openness |  |  |  |  |  |  |
| homophily | 0.07 | 0.35 | 0.837 | **0.47** | **0.22** | **0.033** |
| alter | -0.00 | 0.03 | 0.933 | -0.05 | 0.02 | 0.005 |
| ego | -0.02 | 0.05 | 0.783 | -0.03 | 0.02 | 0.090 |
| Conscientiousness |  |  |  |  |  |  |
| homophily | 0.16 | 0.39 | 0.683 | **-0.64** | **0.21** | **0.002** |
| alter | 0.02 | 0.03 | .0374 | -0.00 | 0.01 | 0.844 |
| ego | **0.12** | **0.05** | **0.010** | 0.02 | 0.02 | 0.292 |
| Extraversion |  |  |  |  |  |  |
| homophily | -0.09 | 0.33 | 0.781 | 0.02 | 0.20 | 0.913 |
| alter | 0.00 | 0.02 | 0.860 | **0.05** | **0.02** | **0.004** |
| ego | 0.00 | 0.03 | 0.902 | -0.01 | 0.02 | 0.469 |
| Agreeableness |  |  |  |  |  |  |
| homophily | -0.19 | 0.42 | 0.649 | 0.46 | 0.29 | 0.109 |
| alter | -0.02 | 0.03 | 0.495 | 0.01 | 0.02 | 0.721 |
| ego | 0.07 | 0.04 | 0.081 | **0.04** | **0.02** | **0.036** |
| Neuroticism |  |  |  |  |  |  |
| homophily | 0.07 | 0.32 | 0.817 | 0.14 | 0.23 | 0.544 |
| alter | -0.02 | 0.02 | 0.478 | **0.05** | **0.02** | **0.003** |
| ego | **0.08** | **0.03** | **0.014** | -0.01 | 0.02 | 0.679 |
| Religion |  |  |  |  |  |  |
| homophily | 0.06 | 0.11 | 0.614 | -0.02 | 0.07 | 0.817 |
| alter | 0.18 | 0.12 | 0.146 | 0.03 | 0.08 | 0.709 |
| ego | -0.22 | 0.18 | 0.214 | 0.01 | 0.08 | 0.930 |
| Political identity |  |  |  |  |  |  |
| homophily | 0.19 | 0.34 | 0.563 | -0.24 | 0.18 | 0.174 |
| alter | -0.07 | 0.05 | 0.232 | 0.08 | 0.04 | 0.074 |
| ego | **0.20** | **0.09** | **0.021** | 0.07 | 0.04 | 0.107 |
| Anxiety |  |  |  |  |  |  |
| homophily | -0.02 | 0.39 | 0.968 | -0.36 | 0.37 | 0.322 |
| alter | -0.01 | 0.02 | 0.836 | 0.00 | 0.02 | 0.972 |
| ego | -0.02 | 0.03 | 0.594 | -0.04 | 0.02 | 0.103 |
| Depression |  |  |  |  |  |  |
| homophily | -0.25 | 0.42 | 0.546 | 0.51 | 0.36 | 0.157 |
| alter | 0.02 | 0.02 | 0.362 | -0.02 | 0.02 | 0.272 |
| ego | -0.01 | 0.03 | 0.695 | **0.04** | **0.02** | **0.016** |
| Cohort popularity |  |  |  |  |  |  |
| homophily | -0.55 | 0.30 | 0.065 | **-0.83** | **0.19** | **<0.001** |
| alter | **-0.02** | **0.01** | **0.020** | **-0.05** | **0.01** | **<0.001** |
| ego | **-0.01** | **0.02** | **0.442** | **-0.02** | **0.00** | **<0.001** |
| Cohort Activity |  |  |  |  |  |  |
| homophily | **-1.38** | **0.32** | **<0.001** | -0.22 | 0.22 | 0.317 |
| alter | **-0.04** | **0.01** | **<0.001** | **-0.04** | **0.01** | **<0.001** |
| ego | **-0.07** | **0.02** | **<.001** | **-0.06** | **0.01** | **<0.001** |
| BEN quality |  |  |  |  |  |  |
| homophily | 0.48 | 0.441 | 0.277 | -0.24 | 0.26 | 0.348 |
| alter | -0.01 | 0.06 | 0.856 | **-0.10** | **0.04** | **0.010** |
| ego | -0.02 | 0.08 | 0.833 | 0.12 | 0.04 | 0.002 |
| BEN quantity |  |  |  |  |  |  |
| homophily | -0.20 | 0.40 | 0.628 | 0.15 | 0.17 | 0.406 |
| alter | -0.00 | 0.00 | 0.423 | -0.01 | 0.01 | 0.301 |
| ego | 0.01 | 0.01 | 0.214 | **0.02** | **0.01** | **<0.001** |

*Note.* BEN denotes baseline ego network; **Bold** denotes significant homophily effects.

Table 4. SIENA estimation results for the long-term for both studies models, with network size as the outcome variable.

| Trait | Study 1 | | | Study 2 | | |
| --- | --- | --- | --- | --- | --- | --- |
|  | **Odds ratio** | **SE** | ***p*** | **Odds ratio** | **SE** | ***p*** |
| Structural Controls |  |  |  |  |  |  |
| outdegree (density) | **-3.54** | **0.20** | **<0.001** | **-3.91** | **0.21** | **<0.001** |
| reciprocity | **1.59** | **0.10** | **<0.001** | **1.43** | **0.07** | **<0.001** |
| transitive triplets | **0.13** | **0.01** | **<0.001** | **0.16** | **0.01** | **<0.001** |
| transitive ties | **1.11** | **0.18** | **<0.001** | **1.21** | **0.21** | **<0.001** |
| Gender |  |  |  |  |  |  |
| homophily | 0.08 | 0.05 | 0.110 | 0.02 | 0.05 | 0.633 |
| alter | -0.10 | 0.08 | 0.184 | -0.02 | 0.06 | 0.726 |
| ego | 0.08 | 0.07 | 0.266 | **0.14** | **0.06** | **0.027** |
| Ethnicity |  |  |  |  |  |  |
| homophily | 0.05 | 0.07 | 0.499 | 0.07 | 0.06 | 0.290 |
| alter | 0.09 | 0.08 | 0.273 | **-0.19** | **0.09** | **0.026** |
| ego | 0.01 | 0.08 | 0.893 | -0.10 | 0.08 | 0.203 |
| School background |  |  |  |  |  |  |
| homophily | 0.03 | 0.05 | 0.546 | 0.04 | 0.05 | 0.413 |
| alter | 0.12 | 0.06 | 0.064 | -0.08 | 0.06 | 0.236 |
| ego | -0.07 | 0.07 | 0.309 | **0.20** | **0.06** | **0.001** |
| Openness |  |  |  |  |  |  |
| homophily | 0.18 | 0.15 | 0.237 | 0.10 | 0.15 | 0.512 |
| alter | **-0.05** | **0.02** | **0.002** | 0.00 | 0.01 | 0.960 |
| ego | -0.01 | 0.02 | 0.410 | -0.02 | 0.01 | .091 |
| Conscientiousness |  |  |  |  |  |  |
| homophily | 0.26 | 0.17 | 0.129 | **-0.32** | **0.14** | **0.024** |
| alter | -0.02 | 0.01 | 0.171 | 0.00 | 0.01 | 0.968 |
| ego | **0.05** | **0.01** | **<0.001** | 0.01 | 0.01 | 0.137 |
| Extraversion |  |  |  |  |  |  |
| homophily | -0.13 | 0.15 | 0.397 | 0.18 | 0.14 | 0.196 |
| alter | 0.00 | 0.01 | 0.744 | **0.03** | **0.01** | **0.006** |
| ego | **0.03** | **0.01** | **0.006** | -0.01 | 0.01 | 0.254 |
| Agreeableness |  |  |  |  |  |  |
| homophily | -0.27 | 0.19 | 0.159 | 0.04 | 0.19 | 0.835 |
| alter | **0.04** | **0.02** | **0.010** | 0.02 | 0.01 | 0.123 |
| ego | **0.04** | **0.02** | **0.039** | **0.04** | **0.01** | **0.001** |
| Neuroticism |  |  |  |  |  |  |
| homophily | -0.18 | 0.15 | 0.217 | 0.06 | 0.15 | 0.719 |
| alter | **-0.02** | **0.01** | **0.050** | **0.02** | **0.01** | **0.019** |
| ego | **0.03** | **0.01** | **0.029** | 0.00 | 0.01 | 0.736 |
| Religion |  |  |  |  |  |  |
| homophily | 0.01 | 0.05 | 0.814 | 0.06 | 0.05 | 0.260 |
| alter | 0.07 | 0.06 | 0.281 | -0.02 | 0.06 | 0.754 |
| ego | **-0.24** | **0.06** | **<0.001** | -0.05 | 0.06 | 0.376 |
| Political Identity |  |  |  |  |  |  |
| homophily | -0.12 | 0.16 | 0.456 | 0.02 | 0.13 | 0.868 |
| alter | 0.04 | 0.03 | 0.118 | **0.08** | **0.03** | **0.012** |
| ego | 0.04 | 0.03 | 0.193 | 0.03 | 0.03 | 0.350 |
| Anxiety |  |  |  |  |  |  |
| homophily | 0.06 | 0.18 | 0.715 | 0.08 | 0.22 | 0.733 |
| alter | 0.02 | 0.01 | 0.074 | -0.03 | 0.01 | 0.051 |
| ego | -0.02 | 0.01 | 0.041 | -0.01 | 0.01 | 0.507 |
| Depression |  |  |  |  |  |  |
| homophily | 0.38 | 0.24 | 0.114 | -0.09 | 0.24 | 0.698 |
| alter | -0.01 | 0.01 | 0.588 | 0.02 | 0.01 | 0.166 |
| ego | 0.01 | 0.01 | 0.167 | 0.02 | 0.01 | 0.108 |
| Cohort Popularity |  |  |  |  |  |  |
| homophily | 0.17 | 0.15 | 0.281 | **-0.58** | **0.15** | **<0.001** |
| alter | -0.01 | 0.01 | 0.221 | **-0.04** | **0.00** | **<0.001** |
| ego | **-0.02** | **0.01** | **0.001** | **-0.02** | **0.00** | **<0.001** |
| Cohort activity |  |  |  |  |  |  |
| homophily | **-0.80** | **0.16** | **<0.001** | **-0.37** | **0.16** | **0.017** |
| alter | **-0.04** | **0.01** | **<0.001** | **-0.05** | **0.01** | **<0.001** |
| ego | **-0.04** | **0.01** | **<0.001** | **-0.05** | **0.01** | **<0.001** |
| BEN quality |  |  |  |  |  |  |
| homophily | 0.33 | 0.19 | 0.080 | -0.12 | 0.18 | 0.511 |
| alter | -0.05 | 0.03 | 0.149 | **-0.11** | **0.03** | **<0.001** |
| ego | **-0.07** | **0.03** | **0.030** | **0.13** | **0.03** | **<0.001** |
| BEN quantity |  |  |  |  |  |  |
| homophily | 0.10 | 0.19 | 0.592 | 0.22 | 0.13 | 0.078 |
| alter | **-0.01** | **0.00** | **0.001** | 0.00 | 0.00 | 0.928 |
| ego | 0.00 | 0.00 | 0.034 | **0.01** | **0.00** | **<0.001** |
| --------------------------------------------------------------------------------------------------------  *Note.* BEN denotes baseline ego network; Bold denotes significant homophily effects. | | | | | | |
